# Time trends in coronary heart disease mortality attributed to outdoor PM2.5 in China: an age-period-cohort analysis using the Global Burden of Disease Study 2019

**DOI:** 10.3389/fpubh.2025.1517507

**Published:** 2025-03-05

**Authors:** Yuan Ma, Yuxiang Huang, Li Li, Li Yu, Pei Xiao, Qian Wang

**Affiliations:** ^1^Department of Medical Record Management, West China Second University Hospital, Sichuan University, Chengdu, Sichuan, China; ^2^Key Laboratory of Birth Defects and Related Diseases of Women and Children (Sichuan University), Ministry of Education, Chengdu, Sichuan, China; ^3^Medical Insurance Office, West China Fourth University Hospital, Sichuan University, Chengdu, Sichuan, China; ^4^Department of Gynecology, West China Second University Hospital, Sichuan University, Chengdu, Sichuan, China

**Keywords:** outdoor PM2.5, coronary heart disease, mortality trends, age-period-cohort model, China

## Abstract

**Background:**

In China, coronary heart disease (CHD) is a significant public health issue affecting the population's health. Evidence suggests that outdoor PM2.5 is a crucial environmental risk factor for CHD mortality. This study aims to provide scientific evidence for the prevention and treatment of CHD by analyzing the trend of CHD mortality attributed to outdoor PM2.5 in China from 1994 to 2019.

**Methods:**

Data were obtained from the Global Burden of Disease Study (GBD) 2019. CHD mortality attributed to outdoor PM2.5 in China from 1994 to 2019 was extracted from the GBD Data tool. We used an age-period-cohort (APC) model based on the intrinsic estimator (IE) algorithm to decompose the age, period, and cohort effects related to CHD mortality attributed to outdoor PM2.5.

**Results:**

From 1994 to 2019, the crude mortality rates (CMRs) and age-standardized mortality rates (ASMRs) of CHD attributed to outdoor PM2.5 in China showed an overall upward trend. The APC model analysis showed that the relative risk of CHD mortality attributed to outdoor PM2.5 increased exponentially with age, reaching 89.284 (95% CI: 48.669, 163.793) in the 90–95 age group. The period effect increased monotonically, with a relative risk of 3.699 (95% CI: 3.639, 3.760) in 2019. The cohort effect decreased monotonically, with the lowest relative risk of CHD mortality attributed to outdoor PM2.5 in residents born between 1990 and 1994, at 0.135 (95% CI: 0.031, 0.588).

**Conclusion:**

The older adult, a high-risk population, should receive more attention. In the future, continuous efforts should be made to strengthen environmental air pollution control and implement targeted health interventions to reduce the impact of outdoor PM2.5 on CHD mortality.

## 1 Introduction

Coronary heart disease (CHD), also known as ischemic heart disease, is a chronic cardiovascular disease typically caused by atherosclerosis of the coronary arteries, which leads to narrowing or even occlusion of coronary arteries, resulting in myocardial ischemia, hypoxia, or necrosis (1, 2). As a typical cardiovascular disease (CVD), CHD is a leading cause of death worldwide (3). In 2015, there were ~400 million cases of CVD worldwide, with CHD accounting for 27% of cases (4). The American Heart Association predicts that the number of CHD cases worldwide may double by 2030 (5). In China, CHD is the second leading cause of death among residents, with nearly 20% of deaths attributable to CHD (6). According to the latest data, there are currently 11.39 million cases of CHD in China, with hospitalization costs reaching nearly 125.6 billion yuan (7). CHD has imposed a heavy medical burden on Chinese families and society. Nowadays, with the increasing aging population, CHD has become one of the important public health issues affecting residents' health.

Many previous studies have shown that particulate matter (PM) with an aerodynamic diameter ≤2.5 μm (PM2.5) is an important environmental risk factor for CHD incidence and mortality (8–11). The impact of PM2.5 on cardiovascular health of residents should not be ignored. By analyzing data on air pollution and CHD cases in Shanghai from 2006 to 2011, Dai et al. (12) found that for every 10 μg/m^3^ increase in PM2.5 concentration, the CHD mortality rate increased by 0.68%. Liu et al. (13) analyzed the spatial and temporal trends of the health effects of air pollution in China from 2004 to 2012 and found that in the areas with high concentrations of PM2.5, such as the Beijing-Tianjin-Hebei region, the Yangtze River Delta, and the Sichuan Basin, PM2.5 exposure levels were positively associated with CHD mortality. In addition, a study of 272 representative cities in China also found that for every 10 μg/m^3^ increase in the 2-day moving average concentration of PM2.5, the CHD mortality rate increased by 0.30% (14).

However, there are some major problems with these epidemiologic studies, which ignore patient age, period and cohort effects, and do not portray well the influence of age, period and cohort effects on changes in the association between PM2.5 and CHD risk. To address these limitations, this study employs an age-period-cohort (APC) model to decompose the age, period, and cohort effects of CHD mortality attributed to outdoor PM2.5 from the data. We also analyze the long-term trends of CHD mortality attributed to outdoor PM2.5. Our findings will contribute to a better understanding of the epidemiology of CHD and provide a scientific basis for the effective prevention and treatment of CHD.

## 2 Methods

### 2.1 Data source

The research data was sourced from the Global Health Data Exchange (GHDx) (15), utilizing the latest Global Burden of Disease Study (GBD) 2019 data for analysis. The database comprehensively assessed the incidence, mortality, and disease burden of 369 diseases and injuries across 204 countries and regions (16). The original data for CHD mortality in China was primarily obtained from the Chinese Center for Disease Control and Prevention (CDC) Cause of Death Reporting System, Disease Surveillance Points (DSPs), and Maternal and Child Health Surveillance Network (17). CHD was diagnosed and identified according to the clinical criteria of the World Health Organization and the classification criteria of the International Classification of Diseases Version 10 (ICD-10).

Estimates of ambient particulate matter pollution exposure came from multiple sources, including satellite observations of aerosols in the atmosphere, ground-based measurements, chemical transport model simulations, population estimates, and land use data (18). In the GBD study, ambient particulate matter pollution was defined as the population-weighted annual mean mass concentration of outdoor PM2.5 exposure (19). GBD 2019 estimated long-term ambient particulate matter pollution exposure by combining satellite data, chemical transport model simulation data with land-use information, and calibrating satellite measurements with ground-based measurements using an air quality data integration model.

To obtain data on CHD mortality attributed to ambient particulate matter. First, GBD 2019 used population attributable fraction (PAF) to obtain the number of CHD deaths attributed to ambient particulate matter. This was then converted to a mortality rate and standardized rates were calculated based on the global standard population. The PAF represents the theoretical minimum risk exposure level (TMREL) for ambient particulate matter. In GBD 2019, the TMREL for ambient particulate pollution was located at 2.4–5.9 μg/m^3^ (20).

According to the relevant procedures of the GHDx database, we extracted CHD mortality data attributed to outdoor PM2.5 in China from 1994 to 2019 (21). The indicators included crude mortality rates (CMRs) for all age groups and 25–94 year olds in 5-year age groups, as well as age-standardized mortality rates (ASMRs). It should be noted that age groups below 25 and above 94 were excluded. This is because the database lacks data for age groups below 25, and the age group above 94 belongs to the open interval, which does not meet the basic requirements of the APC model.

### 2.2 Age-period-cohort model

CHD mortality attributed to outdoor PM2.5 not only reflects the risk of death experienced by residents in a given year but also accumulates the health risks they have faced since birth (22). However, conventional statistical analysis methods were ineffective in decomposing these death risks and health risks (23). The APC model, as a popular statistical tool, can effectively solve this problem. We used the APC model to extract information hidden in age-adjusted mortality rates to estimate the age, period, and cohort effects of CHD mortality attributed to outdoor PM2.5. This allowed us to observe the independent effects of age, period, and birth cohort on time trends in CHD mortality attributed to outdoor PM2.5. The age effects represent the risks associated with residents' physiological aging. The period effects represent changes in society, economy, and culture that lead to similar changes in residents' lives during the same period. The cohort effects represent differences in lifestyle and exposure to risk factors, and residents born in the same year have similar experiences in the same year (24, 25). The general form of the APC model is:


(1) $$\begin{array}{l} M=\gamma +\delta X_{a}+\rho X_{p}+\tau X_{c}+\varepsilon \end{array}$$

In the equation, *M* represents the CHD mortality attributed to outdoor PM2.5, while *X*_*a*_, *X*_*p*_, and *X*_*c*_ represent age, period, and birth cohort, respectively. δ, ρ, and τ denote the age, period, and birth cohort effects, and γ represents the constant term. ε represents the residual.

The traditional APC model could not avoid the problem of collinearity, making it difficult to accurately estimate the net effects of each age group, period, and birth cohort (26). To address this issue, Fu and Yang proposed the Intrinsic Estimator (IE) algorithm (27, 28). This method has the characteristics of estimability and unbiasedness, which can make the coefficients estimated by the APC model robust and reliable. Therefore, we introduced the IE algorithm based on the traditional APC model. The interference of other factors was excluded.

### 2.3 Statistical methods

This study employed Stata 14.0 software (StataCorp, College Station, TX, USA) to conduct APC model analysis, estimating coefficients for age, period, and cohort effects, with statistical significance defined as *p* < 0.05. Deviance, Akaike Information Criterion (AIC), and Bayesian Information Criterion (BIC) were used to evaluate model fit (22). Finally, the exponential value of the coefficient [exp (coef) = *e*^coef^] was calculated to represent the relative risk (RR) of mortality for a given age, period, and birth cohort compared to the average mortality rate (18).

## 3 Results

### 3.1 The overall trend in CHD mortality attributed to outdoor PM2.5

**Figure 1:**
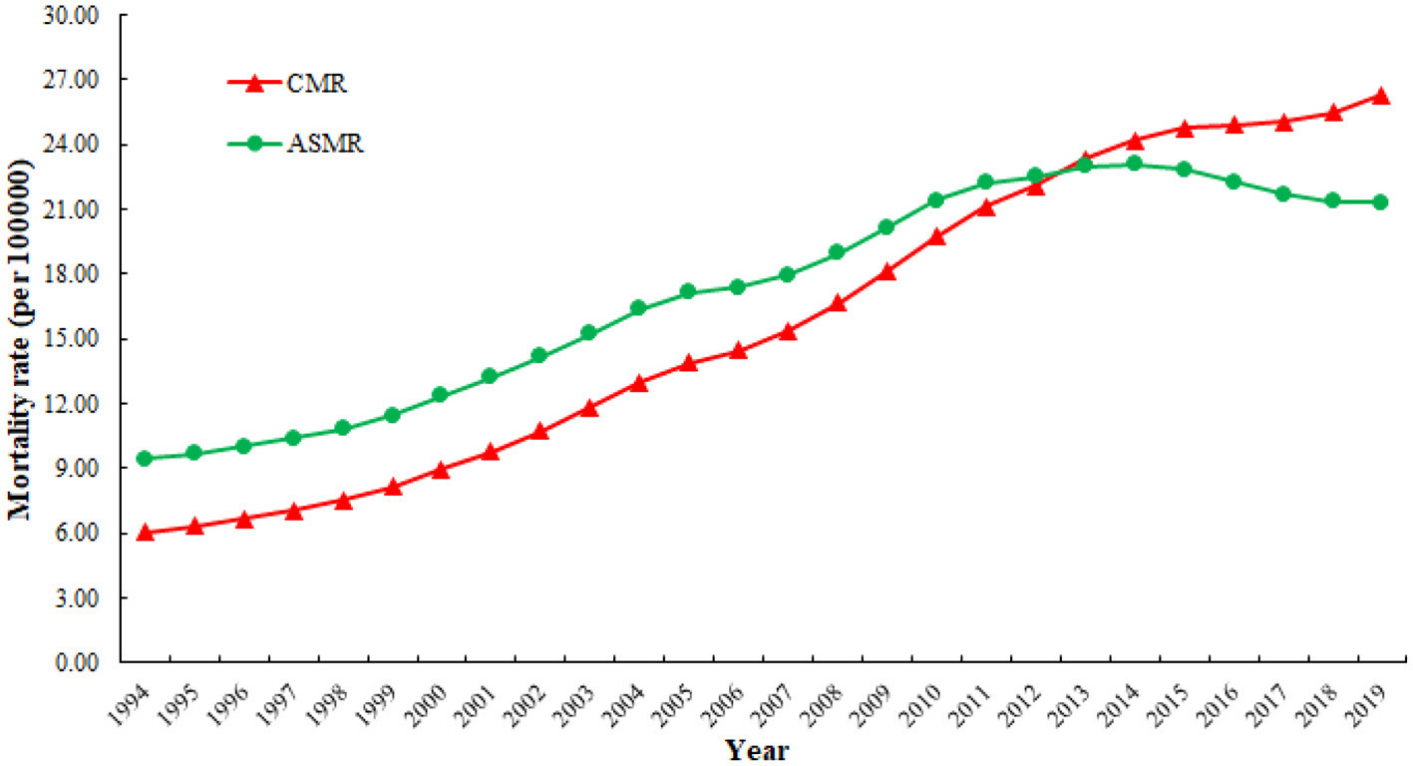
Trends in crude mortality rates (CMRs) and age-standardized mortality rates (ASMRs) of CHD attributed to outdoor PM2.5 from 1994 to 2019.

Figure 1 illustrates the trends in CMRs and ASMRs of CHD attributed to outdoor PM2.5 in China from 1994 to 2019. Overall, ASMRs were higher than CMRs during the period from 1994 to 2012, while CMRs surpassed ASMRs from 2013 to 2019. ASMRs significantly increased from 1994 to 2014, followed by a slight decline from 2014 to 2019. In contrast, CMRs have shown an overall upward trend since 1994.

### 3.2 Age, period, and cohort variations in CHD mortality attributed to outdoor PM2.5

**Figure 2:**
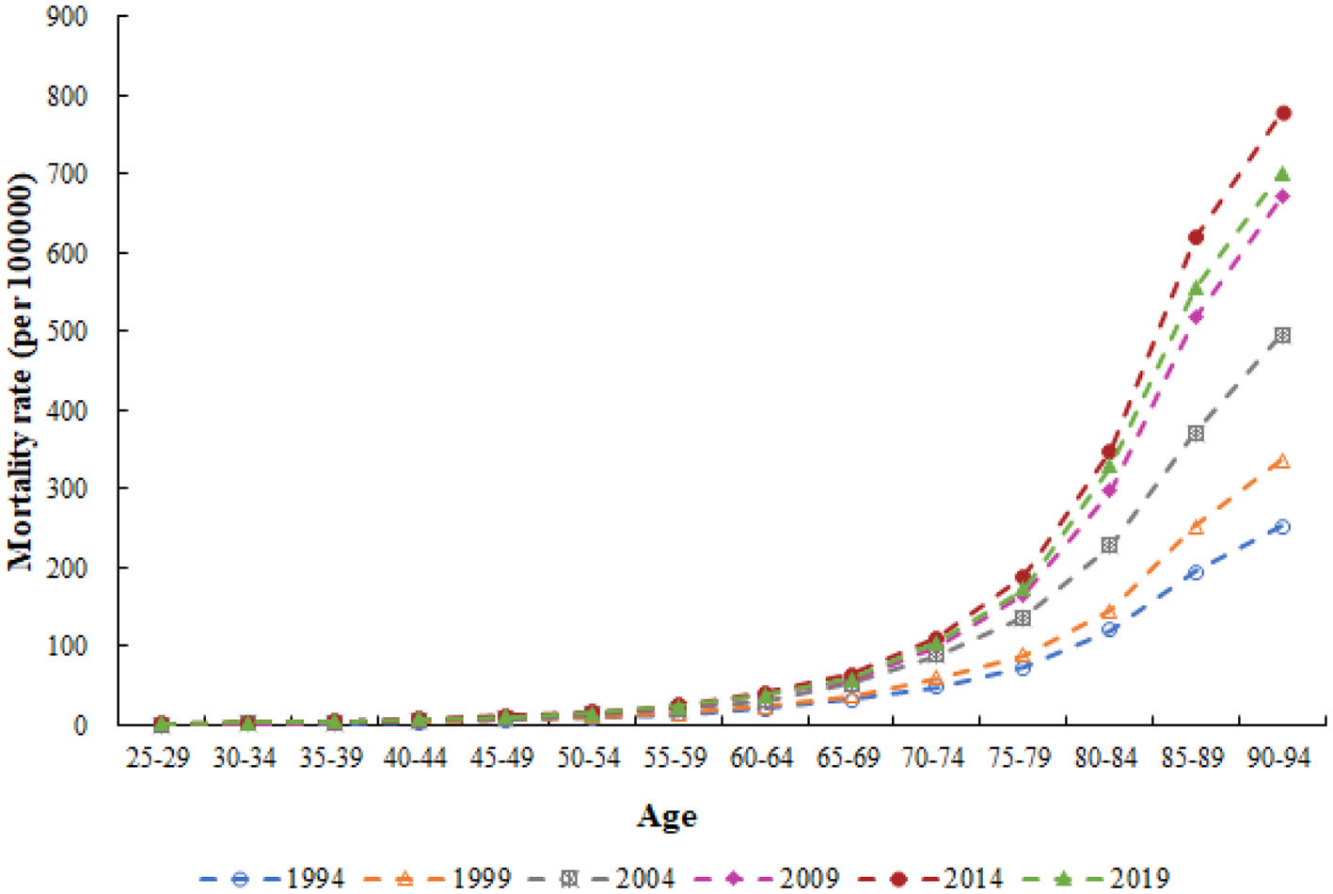
Trends of age-specific CHD mortality attributed to outdoor PM2.5 in China from 1994 to 2019.

Figure 2 shows the trends of age-specific CHD mortality attributed to outdoor PM2.5 in 1994, 1999, 2004, 2009, 2014, and 2019. The CHD mortality attributed to outdoor PM2.5 exhibited an exponential distribution with age, with a significantly higher rate observed in the older adult population. Notably, we observed an increasing trend in CHD mortality attributed to outdoor PM2.5 over the six periods from 1994 to 2019, with a greater increase in mortality rate observed in the older adult population compared to the younger population.

**Figure 3:**
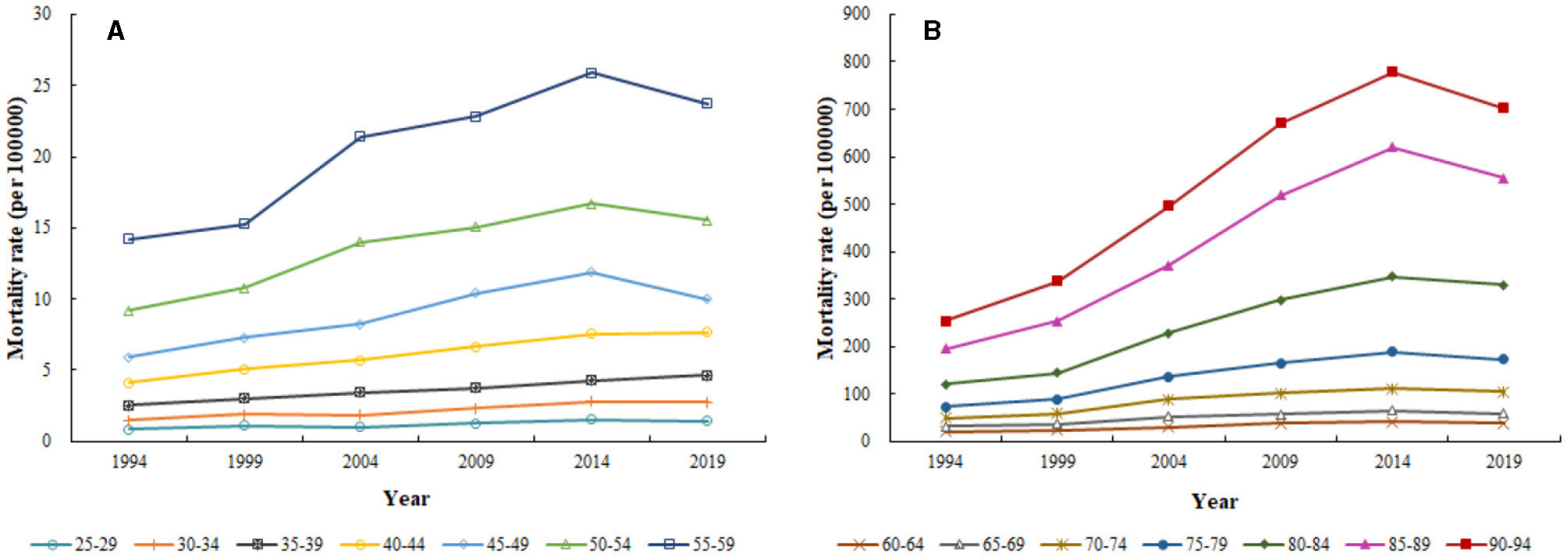
Period-based variation of age-specific CHD mortality attributed to outdoor PM2.5 from 1994 to 2019. **(A)** 25–59 years; **(B)** 60–94 years.

Figure 3 illustrates the period-based variation of age-specific CHD mortality attributed to outdoor PM2.5 from 1994 to 2019. Overall, CHD mortality attributed to outdoor PM2.5 increased over time across all age groups. However, from 2014 to 2019, a certain degree of decline was observed in all age groups except for the 35–39 and 40–44 age groups, which showed an upward trend. Furthermore, we observed a significant increase in CHD mortality attributed to outdoor PM2.5 with age from 1994 to 2019.

**Figure 4:**
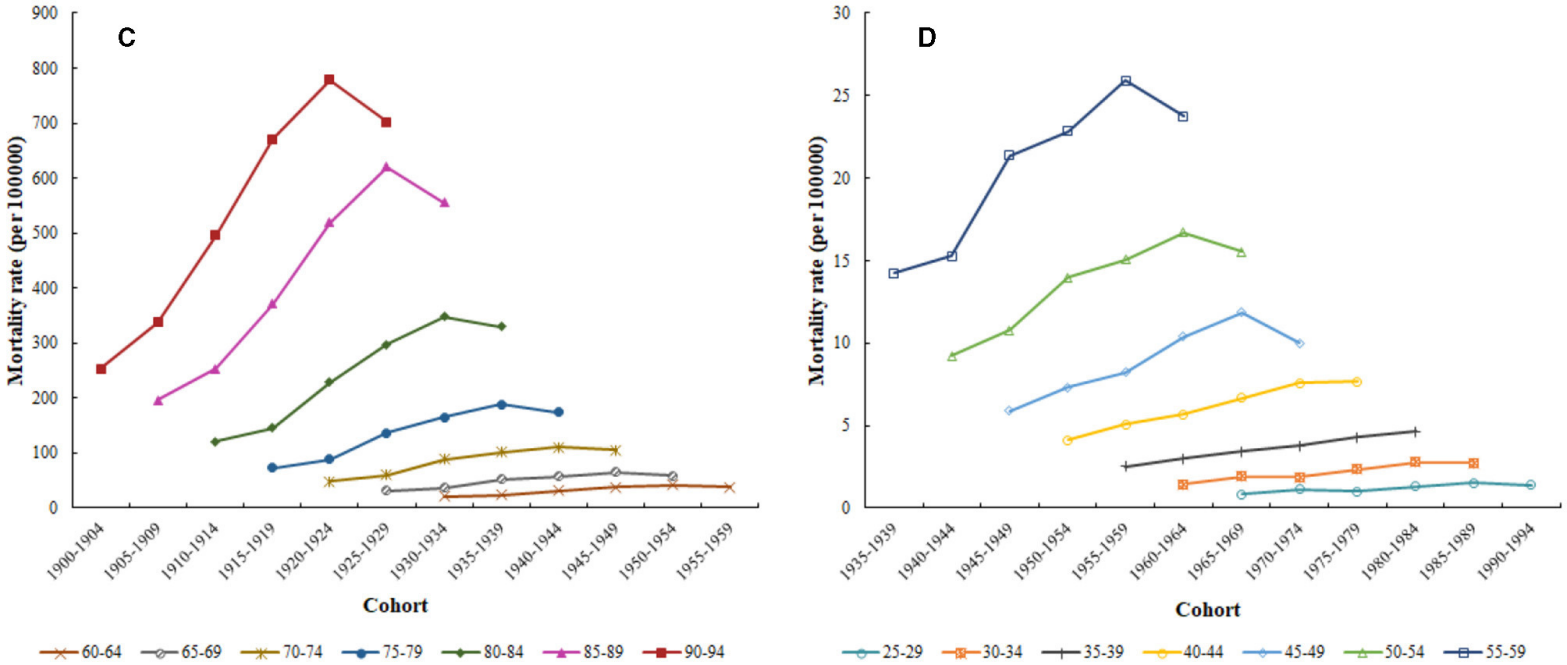
Cohort-based variation of age-specific CHD mortality attributed to outdoor PM2.5 from 1994 to 2019. **(C)** 60–94 years; **(D)** 25–59 years.

Figure 4 shows the cohort-based variation of age-specific CHD mortality attributed to outdoor PM2.5 from 1994 to 2019. Overall, CHD mortality attributed to outdoor PM2.5 increased with the birth year for all age groups, with a greater increase observed in the older adult population. Interestingly, significant differences in CHD mortality attributed to outdoor PM2.5 were observed among individuals of the same age group but with different birth years.

### 3.3 Analysis of APC model on CHD mortality attributed to outdoor PM2.5

Table 1 presents the estimated coefficients, significance levels, 95% confidence intervals, standard errors, and relative risks (RR) for age, period, and cohort effects on CHD mortality attributed to outdoor PM2.5. Regarding age effects, the age effect coefficient for CHD mortality attributed to outdoor PM2.5 increased continuously from −2.249 in the 25–29 age group to 2.243 in the 90–95 age group. The relative risk of death for the 90–95 age group was 89.284 times higher than that for the 25–29 age group. As for period effects, the period effect coefficient increased continuously from −0.785 in 1994 to 0.523 in 2019. The relative risk of death for CHD in 2019 was 3.699 times higher than that in 1994. Concerning cohort effects, the coefficient showed a continuous decreasing trend, decreasing from 0.839 in the 1900–1904 cohort to −1.161 in the 1990–1994 cohort, and the relative risk of death also decreased to 0.135 accordingly. Based on the cohort effect results, we found that people born earlier had a higher mortality rate, while those born later had a lower mortality rate.

**Table 1:** Age-period-cohort (APC) model analysis results of CHD mortality attributed to outdoor PM2.5 in China.

**Variables**	**Coef**	**95% CI**	**S.E**.	**RR**	**95% CI**
**Age**					
25–29	−2.249^***^	−2.991, −1.507	0.378	1.000	
30–34	−1.781^***^	−2.298, −1.264	0.264	1.597	1.275, 1.999
35–39	−1.406^***^	−1.829, −0.984	0.216	2.322	1.688, 3.196
40–44	−1.018^***^	−1.368, −0.667	0.179	3.424	2.316, 5.064
45–49	−0.794^***^	−1.102, −0.486	0.157	4.285	2.776, 6.612
50–54	−0.540^***^	−0.805, −0.274	0.135	5.524	3.430, 8.894
55–59	−0.276^*^	−0.502, −0.050	0.115	7.190	4.292, 12.042
60–64	0.022	−0.164, 0.209	0.095	9.691	5.563, 16.881
65–69	0.329^***^	0.178, 0.480	0.077	13.165	7.291, 23.772
70–74	0.734^***^	0.616, 0.852	0.060	19.746	10.586, 36.835
75–79	1.102^***^	1.004, 1.200	0.050	28.521	14.987, 54.277
80–84	1.584^***^	1.491, 1.678	0.048	46.216	24.175, 88.353
85–89	2.050^***^	1.942, 2.158	0.055	73.585	39.044, 138.684
90–95	2.243^***^	2.108, 2.378	0.069	89.284	48.669, 163.793
**Period**					
1994	−0.785^***^	−0.904, −0.666	0.061	1.000	
1999	−0.500^***^	−0.582, −0.418	0.042	1.330	1.282, 1.379
2004	−0.047	−0.103, 0.008	0.028	2.091	1.962, 2.229
2009	0.291^***^	0.239, 0.342	0.026	2.933	2.741, 3.137
2014	0.519^***^	0.448, 0.591	0.036	3.685	3.515, 3.864
2019	0.523^***^	0.420, 0.625	0.052	3.699	3.639, 3.760
**Cohort**					
1900–1904	0.839^***^	0.604, 1.073	0.120	1.000	
1905–1909	0.815^***^	0.631, 1.000	0.094	0.977	0.929, 1.027
1910–1914	0.763^***^	0.613, 0.912	0.076	0.927	0.851, 1.009
1915–1919	0.710^***^	0.586, 0.833	0.063	0.879	0.787, 0.982
1920–1924	0.663^***^	0.556, 0.769	0.054	0.839	0.738, 0.953
1925–1929	0.589^***^	0.487, 0.692	0.052	0.779	0.683, 0.889
1930–1934	0.511^***^	0.390, 0.631	0.062	0.720	0.643, 0.807
1935–1939	0.417^***^	0.267, 0.567	0.076	0.656	0.603, 0.714
1940–1944	0.249^**^	0.063, 0.434	0.095	0.554	0.528, 0.582
1945–1949	0.126	−0.097, 0.349	0.114	0.490	0.485, 0.496
1950–1954	−0.040	−0.303, 0.223	0.134	0.415	0.404, 0.427
1955–1959	−0.204	−0.507, 0.098	0.154	0.352	0.329, 0.377
1960–1964	−0.360^*^	−0.702, −0.019	0.174	0.302	0.271, 0.336
1965–1969	−0.501^*^	−0.881, −0.122	0.193	0.262	0.227, 0.303
1970–1974	−0.700^**^	−1.140, −0.260	0.224	0.215	0.175, 0.264
1975–1979	−0.808^**^	−1.319, −0.297	0.261	0.193	0.146, 0.254
1980–1984	−0.892^**^	−1.527, −0.256	0.324	0.177	0.119, 0.265
1985–1989	−1.013^*^	−1.894, −0.132	0.449	0.157	0.082, 0.300
1990–1994	−1.161	−2.865, 0.543	0.869	0.135	0.031, 0.588

* *p* < 0.05.

** *p* < 0.01.

*** *p* < 0.001.

Coef, coefficient; S.E., standard error; RR, relative risk; CI, confidence interval; AIC, Akaike information criterions; BIC, Bayesian information criterions.

### 3.4 Age effect and changes in the effect of CHD mortality attributed to outdoor PM2.5

**Figure 5:**
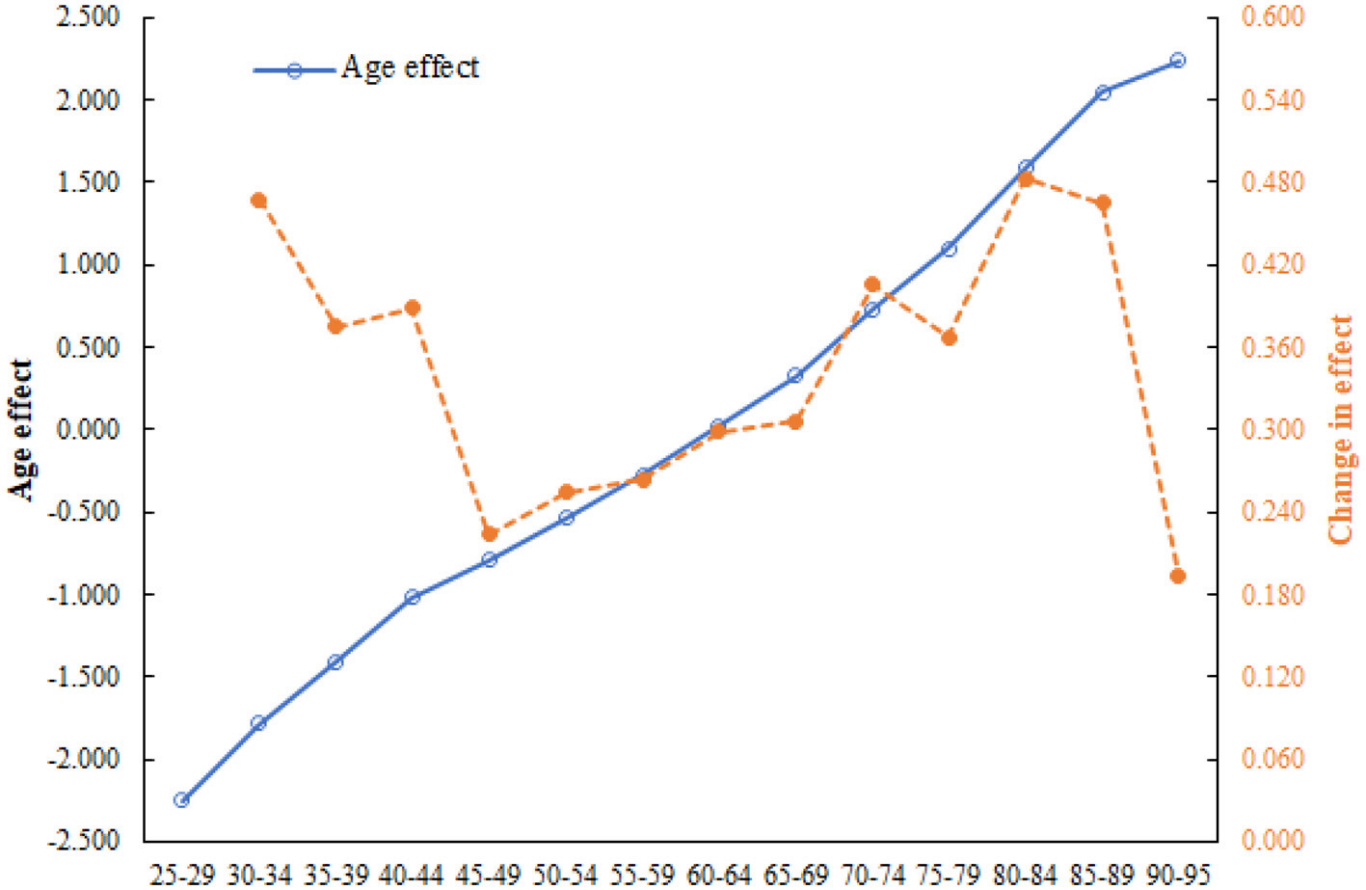
Age effect and changes in the effect of CHD mortality attributed to outdoor PM2.5 in China from 1994 to 2019.

Figure 5 illustrates the age effect (blue line) and changes in the effect (orange line) of CHD mortality attributed to outdoor PM2.5 in China from 1994 to 2019. The age effect curve indicated a gradual increase in CHD mortality attributed to outdoor PM2.5 with advancing age. The age effect changes could be divided into three stages: (1) a rapid decline stage (25–49 years); (2) a slow increase stage (50–84 years); and (3) a steep decline stage (85–95 years).

### 3.5 Period effect and changes in the effect of CHD mortality attributed to outdoor PM2.5

**Figure 6:**
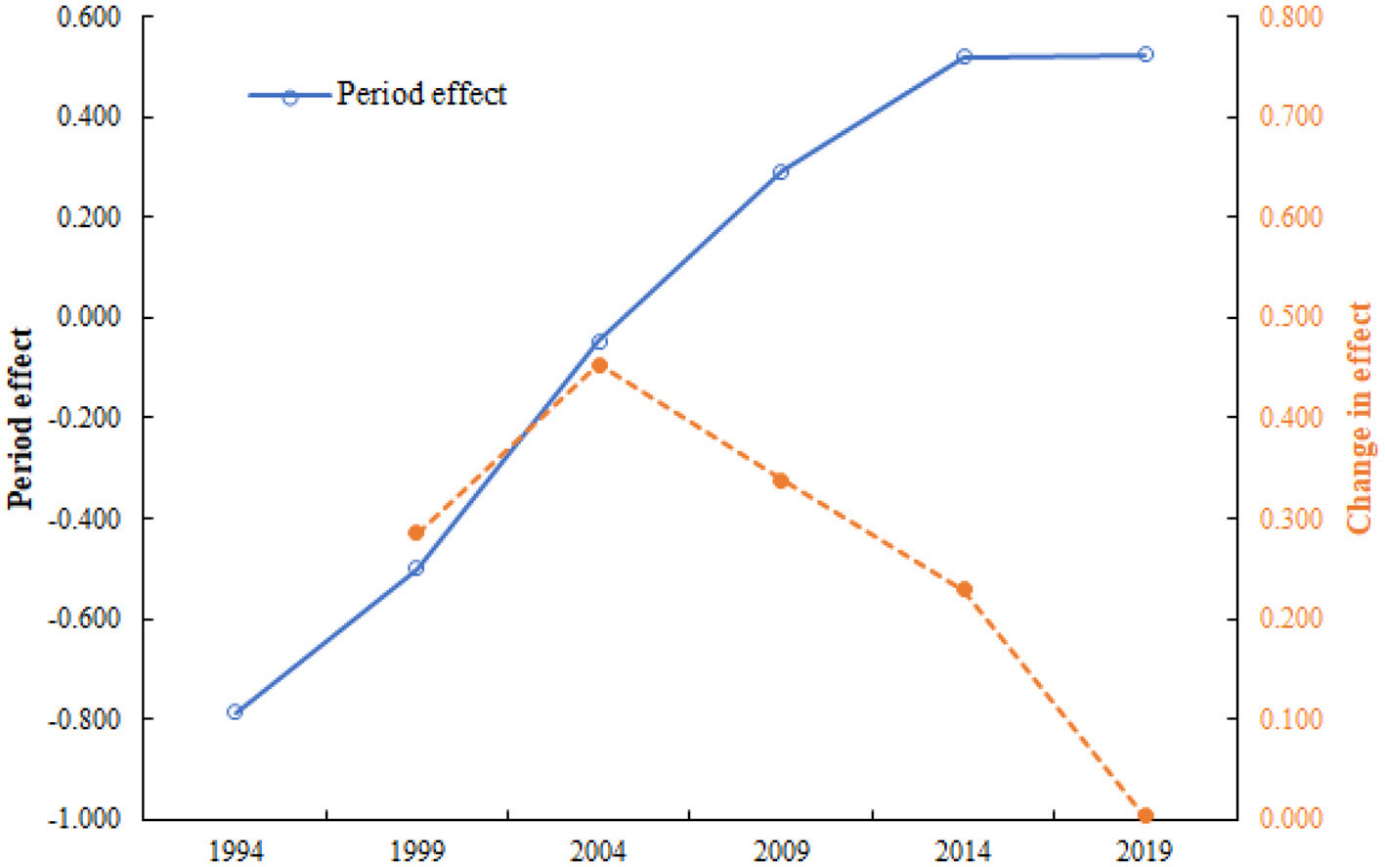
Period effect and changes in the effect of CHD mortality attributed to outdoor PM2.5 in China from 1994 to 2019.

Figure 6 shows the period effect (blue line) and changes in the effect (orange line) of CHD mortality attributed to outdoor PM2.5 in China from 1994 to 2019. Consistent with the changes in age effect, the risk of CHD mortality attributed to outdoor PM2.5 gradually increased over the 25 years. Based on the characteristics of the period effect, the changes in the risk of CHD mortality attributed to outdoor PM2.5 during this period could be divided into two stages: (1) a period of continuous increase (1994–2004); and (2) a period of gradual decline (2004–2019).

### 3.6 Cohort effect and changes in the effect of CHD mortality attributed to outdoor PM2.5

**Figure 7:**
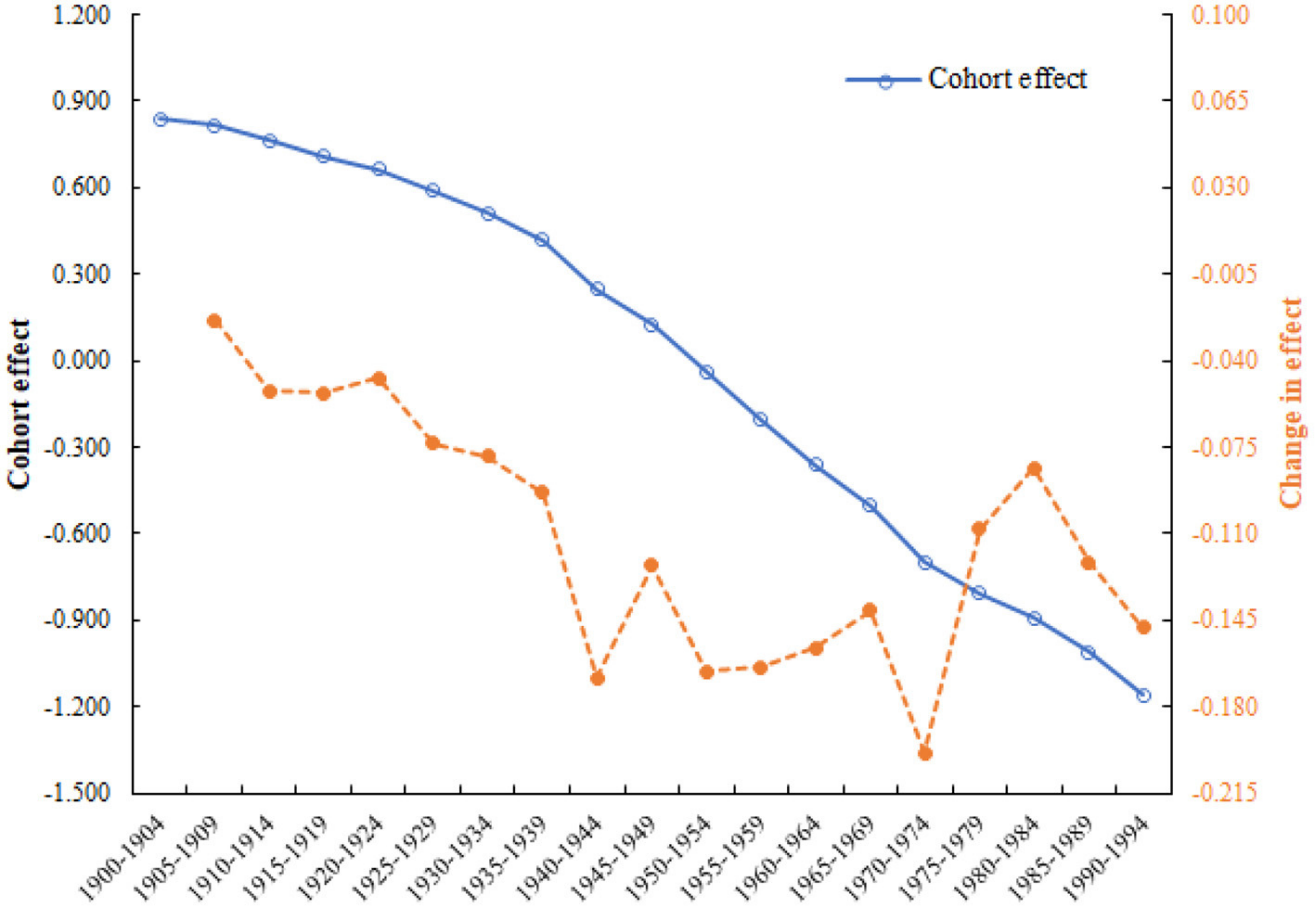
Cohort effect and changes in the effect of CHD mortality attributed to outdoor PM2.5 in China from 1994 to 2019.

Figure 7 displays the estimated cohort effect (blue line) and changes in the effect (orange line). In contrast to the changes in age and period effect, individuals born in later cohorts had a lower risk of CHD mortality attributed to outdoor PM2.5. Based on the characteristics of the cohort effect curve, the changes in the risk of CHD mortality attributed to outdoor PM2.5 could be divided into four stages: (1) gradually declining cohort segment (1900–1944); (2) fluctuating declining cohort segment (1945–1974); (3) rapidly increasing cohort segment (1975–1984); and (4) rapidly declining cohort segment (1985–1994).

## 4 Discussion

With the rapid development of China's economy and society, various regions are facing severe air pollution problems. Epidemiological investigations have shown an association between PM2.5 and CHD mortality (29, 30). Toxicological studies suggest that PM2.5 may cause lung inflammation, cell toxicity, and oxidative stress, leading to an increased risk of cardiovascular death (31–33). In this study, we analyzed the levels and trends of CHD mortality attributed to outdoor PM2.5 in China using GBD 2019 data. By collecting over 25 years of data from 1994 to 2019, we used the APC model to estimate the age, period, and cohort effects of CHD mortality attributed to outdoor PM2.5. The results of this study can reveal the etiology and natural history behind these trend changes, help evaluate the effectiveness of public health policies, identify high-risk groups, and provide data support for the prevention and treatment of CHD in the future.

Our study found that CMRs and ASMRs of CHD attributed to outdoor PM2.5 in China both showed an overall increasing trend from 1994 to 2019. However, the ASMRs decreased slightly after 2014. This was similar to the trend of mortality from all causes of CHD in China analyzed by Wei et al. (34). In addition, a similar validation was obtained in the results of Wang et al. (35), who analyzed the changes in mortality trends of ischemic heart disease in China from 2010 to 2015. The insufficient implementation of public health policies and the limited availability of medical prevention and treatment resources may have contributed to the increase in CHD mortality (35). In contrast, most European countries, the United States, Canada, Australia, and Japan have observed a significant decrease in CHD mortality (36–38). Therefore, we can learn from the experiences and practices of developed countries and formulate appropriate intervention measures suitable for our own country. In addition, a study focusing on Jiangsu Province in China found that from 1990 to 2019, the CHD mortality attributed to PM2.5 showed an overall decrease, while the CHD mortality associated with outdoor air exposure and indoor solid fuel use showed an increase and a decrease, respectively (25). This result indicates regional differences in the trend of CHD mortality changes in China, and outdoor particulate matter should be the focus of current air pollution control efforts.

Consistent with previous studies, age was an important risk factor for CHD (25). This was also shown by Fu et al. (39) in their trend analysis of ischemic heart disease mortality in China from 2010 to 2019. Our study showed that, after controlling for period and birth cohort effects, the risk of CHD mortality attributed to outdoor PM2.5 gradually increased with age. Specifically, the highest risk was observed in the 90–95 age group, which was ~89.284 times higher than that in the 25–29 age group. Age effects mainly reflect physiological changes due to aging and the cumulative effects of exposure to risk factors (40). The risk of CHD mortality attributed to outdoor PM2.5 was higher in the older adult than in the young. On the one hand, physiological changes due to aging led to a decline in immune function in the older adult, making them more susceptible to the effects of outdoor PM2.5 and increasing their risk of death (41, 42). On the other hand, compared to the young, the older adult had weaker health awareness and fewer opportunities to take preventive measures (43). This resulted in the older adult being more exposed to poor sanitary conditions, long-term exposure to outdoor PM2.5, and accumulation of harmful particles in the body (44, 45). In addition, CHD complications were more common in the older adult than in the young, which could also affect CHD mortality in the older adult (46). According to the Seventh National Population Census of China, the proportion of people aged 65 and above reached 13.5% in 2020 (47). China is about to enter a deeply aging society. According to United Nations estimates, by 2050, nearly 400 million people in China will be over 65 years old, of which about 150 million will be over 80 years old (48). This number is surprising, and the burden of CHD mortality attributed to outdoor PM2.5 will be even more severe in the future. Therefore, we should pay attention to the prevention and control of CHD mortality risk in the older adult.

In terms of the period effect, the risk of CHD mortality attributed to outdoor PM2.5 has gradually increased over the past 25 years. Our data showed that the risk of CHD mortality attributed to outdoor PM2.5 in 2019 was ~3.699 times that of 1994. This result was consistent with Wang et al.'s (25) study on the impact of ambient PM on CHD in Jiangsu Province, China. Since the reform and opening up, China's socio-economic growth has been rapid, and urbanization has been gradually accelerating. However, this has led to a deterioration in environmental quality, with increasing atmospheric particulate matter pollution (18, 49). This could explain the trend of increasing risk of CHD mortality attributed to outdoor PM2.5 caused by period effects. It is worth noting that we found a slowdown in the growth rate of CHD mortality attributed to outdoor PM2.5 risk after 2004. The Chinese government issued the “Emission Standards for Particulate Matter from Power Plants” in 2003, proposed more stringent energy-saving and emission reduction policies in 2006, and formulated the Air Pollution Prevention and Control Action Plan and Blue Sky Defense Campaign in 2013 and 2018, respectively (50–52). Therefore, we could conclude that the environmental policies implemented by China in recent years have had a certain effect. Improving air quality could reduce the impact of outdoor PM2.5 on CHD mortality (53). The government should take more measures to address the problem of air pollution and protect public health.

The cohort effect reflects the adverse impact of early exposure to certain unfavorable social-environmental factors on residents' lives, increasing the risk of illness or death (41). Unlike age and period effects, the cohort effect coefficient continuously decreased from the cohort born in 1900–1904 to the cohort born in 1990–1994. Data indicated that the later the birth year of the population, the lower the risk of CHD mortality attributed to outdoor PM2.5. Several studies on CHD have also reached consistent conclusions (24, 38, 54). One possible explanation is that, firstly, China experienced a turbulent society, frequent wars, and negative social and economic productivity in its early stages, leading to a deterioration of living conditions (18). The medical and health conditions in early society were unable to guarantee the health and life of the population (55). Secondly, compared with residents born later, those born earlier were unable to obtain sufficient nutrition during childhood, leading to a weakened immune system to resist diseases (56). Finally, education level was also a critical factor. Residents born earlier had lower education levels and weaker awareness of maintaining health (22). Therefore, they were not aware of the occurrence of CHD or the impact of outdoor PM2.5 on human health (57). When improving CHD prevention and control measures, the government is advised to consider the above factors.

There are several limitations to this study. Firstly, our data source was from GBD 2019. Although GBD 2019 has undergone many corrections and adjustments, including ICD version differences and national variable mapping, as well as the reassignment of incomplete and junk codes, it was difficult to completely avoid bias. Secondly, GBD 2019 did not include CHD mortality attributed to outdoor PM2.5 data in the under-25 age group, and due to the basic requirements of the APC model, the age group above 94 years was excluded from this study. Thirdly, our study lacked an analysis of the risk of CHD mortality attributed to outdoor PM2.5 between urban and rural areas. It was well known that there were significant differences in outdoor PM2.5 exposure levels between urban and rural areas, and the risk of CHD in the population also varied. Of course, it was also extremely important to clarify the risk of CHD mortality in different regions, genders, and occupational groups. In the future, we will conduct a more in-depth analysis of the CHD mortality attributed to outdoor PM2.5.

## 5 Conclusions

This study employed an APC model to evaluate the long-term trends of CHD mortality attributed to outdoor PM2.5 in China from 1994 to 2019. Overall, both CMRs and ASMRs of CHD attributed to outdoor PM2.5 showed an increasing trend from 1994 to 2019. The net effects of age, period, and birth cohort indicated that the risk of CHD mortality attributed to outdoor PM2.5 increased gradually with age and period, but decreased for those born in later years. Therefore, more attention should be paid to the older adult, who are at a higher risk, and efforts to control environmental air pollution should be strengthened.

## Data Availability

Publicly available datasets were analyzed in this study. This data can be found at: https://www.healthdata.org/.

## References

[1] Feng YT Lang CF Chen C Harry Asena M Fang Y Zhang RD. Association between air pollution exposure and coronary heart disease hospitalization in a humid sub-tropical region of China: a time-series study. Front Public Health. (2022) 10:1090443. 10.3389/fpubh.2022.1090443 36711381 PMC9874291

[2] Li J Liu AS. Research progress of CT morphologic and functional evaluation of coronary heart disease. Dis Surveill Control. (2021) 15:406–11. 10.19891/j.issn1673-9388.(2021)05-0406-06

[3] Lozano R Naghavi M Foreman K Lim S Shibuya K Aboyans V. Global and regional mortality from 235 causes of death for 20 age groups in 1990 and 2010: a systematic analysis for the Global Burden of Disease Study 2010. Lancet. (2012) 380:2095–128. 10.1016/S0140-6736(12)61728-0 23245604 PMC10790329

[4] Roth GA Johnson C Abajobir A Abd-Allah F Abera SF Abyu G. Global, regional, and national burden of cardiovascular diseases for 10 causes, 1990 to 2015. J Am Coll Cardiol. (2017) 70:1–25. 10.1016/j.jacc.2017.04.052 28527533 PMC5491406

[5] Benjamin EJ Muntner P Alonso A Bittencourt MS Callaway CW Carson AP. Heart disease and stroke statistics-2019 update: a report from the American Heart Association. Circulation. (2019) 139:e56–e528. 10.1161/CIR.0000000000000659 30700139

[6] Institute for Health Metrics and Evaluation (IHME) GBD Results Tool. (2021). Available online at: http://ghdx.healthdata.org/gbd-results-tool (accessed June 12, 2023).

[7] China Cardiovascular Health and Disease Report Writing Group. Summary of China cardiovascular health and disease report 2021. China J Circ. (2022) 37:553–78.

[8] Zhou M Wang H Zeng X Yin P Zhu J Chen W. Mortality, morbidity, and risk factors in China and its provinces, 1990-2017: a systematic analysis for the Global Burden of Disease Study 2017. Lancet. (2019) 394:1145–58. 10.1016/S0140-6736(19)30427-1 31248666 PMC6891889

[9] Langrish JP Li X Wang S Lee MM Barnes GD Miller MR. Reducing personal exposure to particulate air pollution improves cardiovascular health in patients with coronary heart disease. Environ Health Perspect. (2012) 120:367–72. 10.1289/ehp.1103898 22389220 PMC3295351

[10] Ye X Peng L Kan H Wang W Geng F Mu Z. Acute effects of particulate air pollution on the incidence of coronary heart disease in Shanghai, China. PLoS ONE. (2016) 11:e0151119. 10.1371/journal.pone.0151119 26942767 PMC4778855

[11] Simkhovich BZ Kleinman MT Kloner RA. Particulate air pollution and coronary heart disease. Curr Opin Cardiol. (2009) 24:604–9. 10.1097/HCO.0b013e32833161e5 19696664

[12] Dai J Chen R Meng X Yang C Zhao Z Kan H. Ambient air pollution, temperature and out-of-hospital coronary deaths in Shanghai, China. Environ Pollut. (2015) 203:116–21. 10.1016/j.envpol.2015.03.050 25875162

[13] Liu M Huang Y Ma Z Jin Z Liu X Wang H. Spatial and temporal trends in the mortality burden of air pollution in China: 2004-2012. Environ Int. (2017) 98:75–81. 10.1016/j.envint.2016.10.003 27745948 PMC5479577

[14] Chen R Yin P Meng X Liu C Wang L Xu X. Fine particulate air pollution and daily mortality. A nationwide analysis in 272 Chinese cities. Am J Respir Crit Care Med. (2017) 196:73–81. 10.1164/rccm.201609-1862OC 28248546

[15] Disease GBD Injury I Prevalence C. Global, regional, and national incidence, prevalence, and years lived with disability for 354 diseases and injuries for 195 countries and territories, 1990-2017: a systematic analysis for the Global Burden of Disease Study 2017. Lancet. (2018) 392:1789–858. 10.1016/S0140-6736(18)32279-7 30496104 PMC6227754

[16] Diseases GBD Injuries C. Global burden of 369 diseases and injuries in 204 countries and territories, 1990-2019: a systematic analysis for the Global Burden of Disease Study 2019. Lancet. (2020) 396:1204–22. 10.1016/S0140-6736(20)30925-9 33069326 PMC7567026

[17] Zhou M Wang H Zhu J Chen W Wang L Liu S. Cause-specific mortality for 240 causes in China during 1990-2013: a systematic subnational analysis for the Global Burden of Disease Study 2013. Lancet. (2016) 387:251–72. 10.1016/S0140-6736(15)00551-6 26510778

[18] Liu X Zhou M Yu C Zhang ZJ. Age-period-cohort analysis of type 2 diabetes mortality attributable to particulate matter pollution in China and the US. J Diabetes Res. (2020) 2020:1243947. 10.1155/2020/1243947 32626775 PMC7306083

[19] Chen H Zhou Z Li Z Liang S Zhou J Zou G. Time trends in the burden of stroke and subtypes attributable to PM2.5 in China from 1990 to 2019. Front Public Health. (2022) 10:1026870. 10.3389/fpubh.2022.1026870 36311576 PMC9605206

[20] Collaborators GBDRF. Global burden of 87 risk factors in 204 countries and territories, 1990-2019: a systematic analysis for the Global Burden of Disease Study 2019. Lancet. (2020) 396:1223–49. 10.1016/S0140-6736(20)30752-2 33069327 PMC7566194

[21] Qu Y Wang TY Yang J Zhang J Lv J. Data extraction method and flow of GBD database. China J Evid Based Cardiovasc Med. (2019) 11:1043–6.

[22] Luo L Jiang J Zhang G Wang L Wang Z Yang J. Stroke mortality attributable to ambient particulate matter pollution from 1990 to 2015 in China: an age-period-cohort and spatial autocorrelation analysis. Int J Environ Res Public Health. (2017) 14:772. 10.3390/ijerph14070772 28703768 PMC5551210

[23] Wang P Xu C Yu C. Age-period-cohort analysis on the cancer mortality in rural China: 1990-2010. Int J Equity Health. (2014) 13:1. 10.1186/1475-9276-13-1 24383432 PMC4029464

[24] Wang T Ma Y Li R Sun J Huang L Wang S. Trends of ischemic heart disease mortality attributable to household air pollution during 1990-2019 in China and India: an age-period-cohort analysis. Environ Sci Pollut Res Int. (2022) 29:87478–89. 10.1007/s11356-022-21770-1 35809174

[25] Wang W Zhou N Yu H Yang H Zhou J Hong X. Time trends in ischemic heart disease mortality attributable to PM(2.5) exposure in Southeastern China from 1990 to 2019: an age-period-cohort analysis. Int J Environ Res Public Health. (2023) 20:973. 10.3390/ijerph20020973 36673728 PMC9859070

[26] Robertson C Gandini S Boyle P. Age-period-cohort models: a comparative study of available methodologies. J Clin Epidemiol. (1999) 52:569–83. 10.1016/s0895-4356(99)00033-5 10408997

[27] Fu WJ. Ridge estimator in Singulah Oesiun with application to age-period-cohort analysis of disease rates. Commun Stat Theory Methods. (2000) 29:263–78.

[28] Yang Y Schulhofer-Wohl S Fu WJ Land KC. The intrinsic estimator for age-period-cohort analysis: what it is and how to use it. Am J Sociol. (2008) 113:1697–736. 10.1086/587154

[29] Breitner S Liu L Cyrys J Bruske I Franck U Schlink U. Sub-micrometer particulate air pollution and cardiovascular mortality in Beijing, China. Sci Total Environ. (2011) 409:5196–204. 10.1016/j.scitotenv.2011.08.023 21937089

[30] Ito K Mathes R Ross Z Nadas A Thurston G Matte T. Fine particulate matter constituents associated with cardiovascular hospitalizations and mortality in New York City. Environ Health Perspect. (2011) 119:467–73. 10.1289/ehp.1002667 21463978 PMC3080927

[31] Munzel T Sorensen M Gori T Schmidt FP Rao X Brook FR. Environmental stressors and cardio-metabolic disease: part II-mechanistic insights. Eur Heart J. (2017) 38:557–64. 10.1093/eurheartj/ehw294 27460891 PMC5381593

[32] Schins RP Lightbody JH Borm PJ Shi T Donaldson K Stone V. Inflammatory effects of coarse and fine particulate matter in relation to chemical and biological constituents. Toxicol Appl Pharmacol. (2004) 195:1–11. 10.1016/j.taap.2003.10.002 14962500

[33] Sorensen M Daneshvar B Hansen M Dragsted LO Hertel O Knudsen L. Personal PM25 exposure and markers of oxidative stress in blood. Environ Health Perspect. (2003) 111:161–6. 10.1289/ehp.111-1241344 12573899 PMC1241344

[34] Wei D Xiao W Zhou L Guo J Lu W Wang Y. Age-period-cohort analysis of ischemic heart disease morbidity and mortality in China, 1990-2019. Circ J. (2022) 86:1437–43. 10.1253/circj.CJ-21-0749 35569970

[35] Wang B Li P He F Sha Y Wan X Wang L. Spatiotemporal variations in ischemic heart disease mortality and related risk factors in China between 2010 and 2015: a multilevel analysis. BMC Public Health. (2021) 21:9. 10.1186/s12889-020-10019-6 33397345 PMC7784031

[36] Ford ES Capewell S. Coronary heart disease mortality among young adults in the U.S. from 1980 through 2002: concealed leveling of mortality rates. J Am Coll Cardiol. (2007) 50:2128–32. 10.1016/j.jacc.2007.05.056 18036449

[37] Levi F Chatenoud L Bertuccio P Lucchini F Negri E La Vecchia C. Mortality from cardiovascular and cerebrovascular diseases in Europe and other areas of the world: an update. Eur J Cardiovasc Prev Rehabil. (2009) 16:333–50. 10.1097/HJR.0b013e328325d67d 19369880

[38] Ma E Iso H Takahashi H Yamagishi K Tanigawa T. Age-period-cohort analysis of mortality due to ischemic heart disease in Japan, 1955 to 2000. Circ J. (2008) 72:966–72. 10.1253/circj.72.966 18503224

[39] Fu X Wang J Jiang S Wu J Mu Z Tang Y. Mortality trend analysis of ischemic heart disease in China between 2010 and 2019: a joinpoint analysis. BMC Public Health. (2023) 23:644. 10.1186/s12889-023-15549-3 37016366 PMC10071740

[40] Wen H Xie C Wang L Wang F Wang Y Liu X. Difference in long-term trends in COPD mortality between China and the U.S., 1992(-)2017: an age(-)period(-)cohort analysis. Int J Environ Res Public Health. (2019) 16:1529. 10.3390/ijerph16091529 31052180 PMC6540060

[41] Wang F Mubarik S Zhang Y Wang L Wang Y Yu C. Long-term trends of liver cancer incidence and mortality in China 1990-2017: a joinpoint and age-period-cohort analysis. Int J Environ Res Public Health. (2019) 16:2878. 10.3390/ijerph16162878 31408961 PMC6719938

[42] Qian Y Zhu M Cai B Yang Q Kan H Song G. Epidemiological evidence on association between ambient air pollution and stroke mortality. J Epidemiol Community Health. (2013) 67:635–40. 10.1136/jech-2012-201096 23661720

[43] Hansen S Baptiste KE Fjeldborg J Horohov DW. A review of the equine age-related changes in the immune system: comparisons between human and equine aging, with focus on lung-specific immune-aging. Ageing Res Rev. (2015) 20:11–23. 10.1016/j.arr.2014.12.002 25497559

[44] Wu X Zhu B Zhou J Bi Y Xu S Zhou B. The epidemiological trends in the burden of lung cancer attributable to PM(25) exposure in China. BMC Public Health. (2021) 21:737. 10.1186/s12889-021-10765-1 33858412 PMC8051098

[45] Kanasi E Ayilavarapu S Jones J. The aging population: demographics and the biology of aging. Periodontol 2000. (2016) 72:13–8. 10.1111/prd.12126 27501488

[46] Menezes AR Lavie CJ Forman DE Arena R Milani RV Franklin BA. Cardiac rehabilitation in the elderly. Prog Cardiovasc Dis. (2014) 57:152–9. 10.1016/j.pcad.2014.01.002 25216614

[47] Office of the Leading Group for the Seventh National Census of the State Council. Main data of the Seventh National Census in 2020. (2021). Available online at: http://www.stats.gov.cn/sj/pcsj/rkpc/d7c/202303/P020230301403217959330.pdf (accessed June 14, 2023).

[48] Fang EF Scheibye-Knudsen M Jahn HJ Li J Ling L Guo H. A research agenda for aging in China in the 21st century. Ageing Res Rev. (2015) 24:197–205. 10.1016/j.arr.2015.08.003 26304837 PMC5179143

[49] Li X Song J Lin T Dixon J Zhang G Ye H. Urbanization and health in China, thinking at the national, local and individual levels. Environ Health. (2016) 15(Suppl 1):32. 10.1186/s12940-016-0104-5 26961780 PMC4895783

[50] Jin Y Andersson H Zhang S. Air pollution control policies in china: a retrospective and prospects. Int J Environ Res Public Health. (2016) 13:1219. 10.3390/ijerph13121219 27941665 PMC5201360

[51] Huang J Pan X Guo X Li G. Health impact of China's Air Pollution Prevention and Control Action Plan: an analysis of national air quality monitoring and mortality data. Lancet Planet Health. (2018) 2:e313–23. 10.1016/S2542-5196(18)30141-4 30074894

[52] Yang X Wang Y Chen D Tan X Tian X Shi L. Does the “Blue sky defense war policy” paint the sky blue?-a case study of Beijing-Tianjin-Hebei Region, China. Int J Environ Res Public Health. (2021) 18:2397. 10.3390/ijerph182312397 34886123 PMC8657255

[53] Hall J Zhong J Jowett S Mazzeo A Thomas GN Bryson JR. Regional impact assessment of air quality improvement: the air quality lifecourse assessment tool (AQ-LAT) for the West Midlands combined authority (WMCA) area. Environ Pollut. (2024) 356:123871. 10.1016/j.envpol.2024.123871 38729507

[54] Peltonen M Asplund K. Age-period-cohort effects on ischaemic heart disease mortality in Sweden from 1969 to 1993, and forecasts up to 2003. Eur Heart J. (1997) 18:1307–12. 10.1093/oxfordjournals.eurheartj.a015443 9458424

[55] Gutierrez OM Muntner P Rizk DV McClellan WM Warnock DG Newby PK. Dietary patterns and risk of death and progression to ESRD in individuals with CKD: a cohort study. Am J Kidney Dis. (2014) 64:204–13. 10.1053/j.ajkd.2014.02.013 24679894 PMC4111976

[56] Hankey GJ. Nutrition and the risk of stroke. Lancet Neurol. (2012) 11:66–81. 10.1016/S1474-4422(11)70265-4 22172622

[57] Cohen AK Syme SL. Education: a missed opportunity for public health intervention. Am J Public Health. (2013) 103:997–1001. 10.2105/AJPH.2012.300993 23597373 PMC3698749

